# Age-period-cohort effects in half a century of motor vehicle theft in the United States

**DOI:** 10.1186/s40163-020-00126-5

**Published:** 2020-10-01

**Authors:** Anthony Dixon, Graham Farrell

**Affiliations:** School of Law, University of Leeds, Leeds, LS2 9JT UK

**Keywords:** Age-period-cohort models, S-constraint, Vehicle theft, Security hypothesis, Juvenile crime, Developmental criminology, Developmental crime science

## Abstract

Adopting and refining O’Brien’s S-constraint approach, we estimate age-period-cohort effects for motor vehicle theft offences in the United States for over half a century from 1960. Taking the well-established late-teen peak offending age as given, we find period effects reducing theft in the 1970 s, and period, but particularly cohort effects, reducing crime from the 1990s onwards. We interpret these effects as consistent with variation in the prevailing level of crime opportunities, particularly the ease with which vehicles could be stolen. We interpret the post-1990s cohort effect as triggered by a period effect that operated differentially by age: improved vehicle security reduced juvenile offending dramatically, to the extent that cohorts experienced reduced offending across the life-course. This suggests the prevailing level of crime opportunities in juvenile years is an important determinant of rates of onset and continuance in offending in birth cohorts. We outline additional implications for research and practice.

## Introduction

**Fig. 1:**
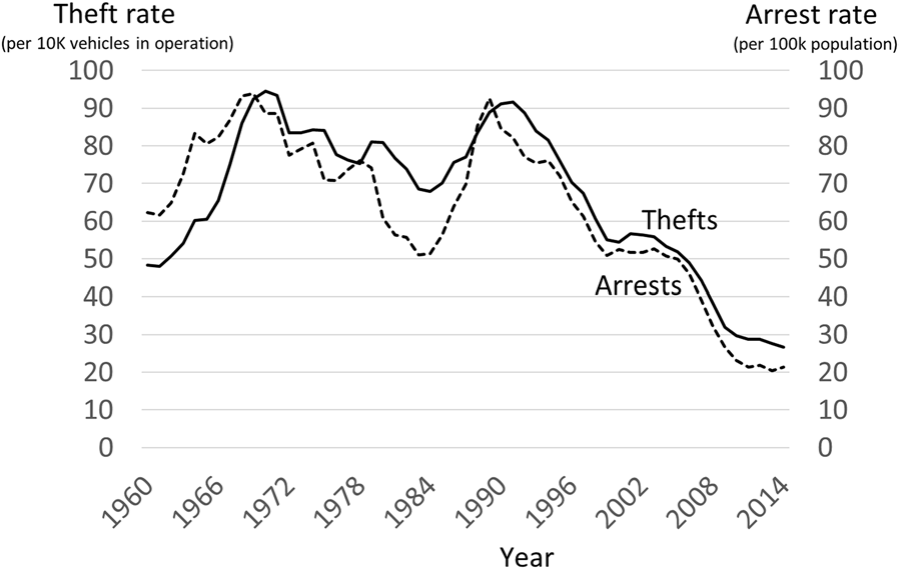
Vehicle thefts per 10,000 vehicles in operation, and vehicle theft arrests per 100,000 population, 1960-2014

The United States dominated the global car market for most of the twentieth century. By the 1950s, over three quarters of the world’s vehicle manufacturing occurred in the US (Heitmann and Morales 2014). While the improved transportation this provided was a feature of rapid economic growth, one of the inadvertent consequences was that there was a rapid increase in vehicle theft between the Second World War and 1960 (President’s Commission 1967). Since then, the trend has varied considerably, as shown in Fig. 1. From 1970 there was a downturn and the rate decreased for 15 years and by over a quarter (28.3 percent) by 1984. A resurgence brought a secondary peak in 1991, but the rate had more than halved by well over two decades of decline by 2014. The main aim of the present study is to shed light on the causes of change in the trend.

Most vehicle theft is reported to the police, so FBI’s Uniform Crime Reports (UCR) used in Fig. 1 are taken to represent actual vehicle theft (President’s Commission 1967, Harlow 1988, Morgan and Truman 2018, Lauritsen et al. 2016). The denominator for the rate was the number of vehicles in operation (from Oak Ridge National Laboratory 2019), which is a better measure of available targets than the number of people in the population (Clarke and Harris 1992).

Our analytic approach is age-period-cohort (APC) analysis, which has been used in numerous disciplines. In the study of cancer, for example, APC analysis provides information about whether disease prevalence is related to the age of patients, when they were born (the cohort), or something that happened at a particular point in time (a period). This is important information because, if it is known whether cancer is related to age, period, or cohort, it helps identify strategies to prevent it.

In the context of offending, and adapting the examples in the definitions of Tonry, Ohlin and Farrington (1991; 30), a*ge effects* are influences on offending that occur with age. For example, rates of offending increase rapidly among juveniles and peak at ages in the late teens, then decline–irrespective of the cohort or period. *Period effects* are influences on offending due to a historical period that are independent of age and cohort effects. For example, if the lockdowns experienced in response to the coronavirus (covid-19) pandemic affected everyone’s likelihood of offending, irrespective of age or birth cohort, that would be a period effect. *Cohort effects* are influences on offending due to membership in one birth cohort rather than another. For example, consider a birth cohort that grew up when crime was easy and rewarding to commit, meaning more individuals became involved and continued in offending, the cohort aging with an unusually high rate of life-course offenders—independent of age or period effects.

The strategic role of APC analysis is to provide information about potential underlying mechanisms of change. It does not reveal precisely what happened, but it can help rule out some possibilities and help identify potential candidate explanations. Amid the larger APC literature, a number of studies relating to crime and criminal justice issues have adopted the approach (including Smith 1986, Steffensmeier, Streifel and Harer 1987, O’Brien 1989, 2002, 2010, 2019a, Steffensmeier, Streifel and Shihadeh 1992, O’Brien, Stockard and Isaacson 1999, Fabio et al. 2006, Smith 2008, O’Brien and Stockard 2009). While APC is the generic term for the approach, there are a variety of methodologies by which it has been undertaken, and it is worth noting the orientation of key reviews in recent years. In the 1980’s, a review concluded that “Given all the documented potential sources for error, the current state-of the-art regarding the modeling of APC data should be considered to be at an early stage of development.” (Kupper et al. 1985; 811). By the 2000’s, a review of the state-of the-art was optimistic in concluding that recent studies “call attention to the multilevel nature of the problem and draw on advances in methods including nonparametric smoothing, fixed and random effects, and identification in structural or causal models.” (Smith, 2008; 287). However, the context of our work is a recent cross-disciplinary review which concluded that “the three effects, and the processes that underlie them, are often misunderstood, and statistical attempts to uncover these effects are often fundamentally flawed to the point that results from such analysis are sometimes devoid of meaning.” (Bell 2020; 208). We discuss our approach later, but refer interested reader’s to Bell’s review, and the work of O’Brien (2014, 2019a, b) in particular, as key influences. We do this in order that the bulk of this study is focused upon providing transparency with respect to our data, assumptions and the details of this specific application of the APC approach.

APC analysis requires age-related data. Arrest data is preferred here, because it has information on the age of offenders, which crime rate data does not. Motor vehicle theft arrests recorded by the FBI and the Bureau of Justice Statistics comprise the main data used in this study. There are fewer arrests than there are vehicle thefts in absolute terms, because not all thefts result in an arrest. However, as Fig. 1 shows, the two measures track each other well over time, and a similar fit was observed between crime and arrest rate trends in a recent study of homicide (O’Brien 2019b) and more generally for different crime types (Farrell 2018). Based on the strength of the relationship between the two sources, from here onwards we assume that the arrest rate trend is representative of the vehicle theft trend. Further specifics on use of the arrest data are given in the methods section below.

### Previous research into vehicle theft trends

One of the benefits of examining motor vehicle theft is a repository of knowledge about the causes of trends in that crime type. An international literature offers strong evidence that changes to vehicle security played a prominent role in vehicle theft trends of recent decades in Australia (Kriven and Ziersch 2007), The Netherlands (van Ours and Vollaard 2016), Germany (Bӓssmann 2011), and the United Kingdom (Farrell and Brown 2016), as well as a systematic review of the effectiveness of the electronic immobilizer (Brown 2015). As motor vehicle security evolved it became increasingly ‘elegant’, that is, ethical and unobtrusive, easy to use, and aesthetically neutral or pleasing (Farrell and Tilley 2020).

The international research is consistent with that for the US. In the post-war period, vehicle theft became increasingly popular among juveniles and young adults who took cars for joyriding and transportation (Harris and Clarke 1991, Clarke and Harris 1992). As theft rates increased, the pressure on vehicle manufacturers to improve security also increased (Newman 2004). Despite resistance from manufacturers (Karmen 1981), the 1968 Motor Vehicle Safety Standard No. 114 required the installation during manufacture of improved door locks, steering wheel ignition locks, and buzzers to alert drivers who left the key in the ignition. These measures were implemented by 1969, and they, but the steering wheel ignition lock in particular, are held responsible for halting the increase in vehicle thefts and inducing the decline shown in Fig. 1 across the 1970s and early 1980s (Webb 1994). From 1984, vehicle theft resumed a steep upward trend: steering wheel ignition locks could be broken and cars ‘hotwired’, and with many new and attractive sporty vehicles on the market, the national rate achieved a further peak in 1991. During this period, the 1984 Motor Vehicle Theft Law Enforcement Act was passed, requiring manufacturers to introduce parts-marking for 14 different components of vehicles defined as high-risk (those exceeding the 1983-84 median theft rate). Vehicle lines could be exempted from parts marking if they were fitted with improved anti-theft devices, of which the electronic immobilizer was the most prominent and effective. The e-immobilizer was introduced from the late 1980s (National Highway Traffic Safety Administration 1998, Brown 2013, Fujita and Maxfield 2012), and a gradual decline in vehicle theft across the 1990s occurred as new secure vehicles replaced old ones on the road. In the years that followed, continued refinements to vehicle security occurred in parallel to offender efforts to circumvent them in an ‘evolutionary arms race’ (Brown 2017). In the 2000s, some older less secure vehicles continued to be stolen for transportation and scrap, particularly late 1990s Honda saloons which regularly topped the most-stolen-vehicle charts, while more expert offenders circumvented the security on luxury vehicles that could be chopped for parts or resold (Barro 2014).

This was an overview of what we consider to be strong evidence that improved vehicle security played a key role in the declines in vehicle theft both in the 1970s and from the 1990s onwards. The present study is concerned with whether or not APC effects are consistent with this previous research. We find that they are consistent. Importantly, however, we also find that APC analysis facilitates significant new insight into the nature of events and, potentially, into the nature of crime and offending more generally.

## Data and method

Aspects of the data and method were introduced above. This section provides detail.

### The arrest data

The data are annual UCR age-related arrest rates for vehicle theft 1960 to 2014. Those for 1980 to 2014 were obtained from the Bureau of Justice Statistics (BJS) online data arrest tool (Snyder, Cooper and Mulako-Wangota 2017), and those for 1960 to 1979 were received as photocopies of datasheets sent to us by the FBI (see acknowledgements). The BJS arrest data included population and rates, and population data from the Census Bureau was used to calculate rates for 1960-79. As noted in O’Brien (2019a), arrest data from the earlier years is from a smaller sample of reporting agencies than the later years, so a smaller proportion of the population is covered. This may mean a greater error factor in earlier years, but there is no reason to suspect this distorts the overall findings or their interpretation. O’Brien's (2019a) homicide research showed that adjusting the data for clearance rates made little or no difference, and so unadjusted data is preferred here.

We undertook further quality control. Data for the 4 years 1960–1963 had missing values for the very young (Under 13) and the moderately old (ages 55 +). We estimated these missing values using the distribution from nearby years on the basis that they would add value while any error introduced to the study’s overall findings and their interpretation would be small. The printed datasheets received from the FBI had some data entry errors for 1973. There were implausible values for some arrest counts, and some data appeared displaced within the year on the printed sheets, so we used a population-weighted average from contiguous years to replace the corrupted data.

The data were processed into a data matrix with age and period on the main axis and cohorts on the diagonals. The product, grouped into 5-year periods and 5-year age groups, is shown as Table 1.

**Table 1:** Age-period specific motor vehicle theft rates per 100,000 US residents

Age	Period										
	1960–1964	1965–1969	1970–1974	1975–1979	1980–1984	1985–1989	1990–1994	1995–1999	2000–2004	2005–2009	2010 -2014
Und15	34.07	45.57	40.76	37.92	64.12	107.00	139.80	85.97	54.13	31.54	13.66
15–19	509.47	565.78	465.07	396.05	279.73	420.13	512.23	361.99	264.72	173.13	83.81
20–24	136.69	155.20	155.92	134.75	127.68	171.34	178.13	145.34	143.99	105.64	58.38
25–29	57.20	70.14	77.78	68.97	70.82	98.59	103.26	87.29	86.03	74.06	47.06
30–34	33.31	39.02	47.26	40.41	42.68	62.41	68.01	66.59	66.35	55.40	38.58
35–39	22.68	24.80	30.83	27.42	27.60	37.14	41.46	45.77	51.03	44.76	27.68
40–44	14.33	16.34	18.83	17.80	18.47	21.48	23.23	26.30	32.09	33.22	20.61
45–49	8.15	9.61	11.78	11.34	12.04	12.47	12.68	13.81	16.84	19.72	14.34
50–54	7.41	5.36	6.35	6.63	6.77	7.36	6.96	6.67	7.70	9.19	7.99
55–59	2.31	2.78	3.41	3.54	4.11	3.98	3.52	3.50	3.51	3.94	3.65
60–64	1.18	1.19	1.66	1.69	1.93	1.88	1.73	1.77	1.57	1.79	1.48

(Source: FBI, Bureau of Justice Statistics)

Table 1 is constructed by age and period, which means that cohorts can be traced along the diagonals. For example, the cohort born around 1970 enters the table as a cohort in 1985–1989 as 15 to 19-year olds with a motor vehicle theft rate of 420.13. Following the diagonal down and to the right tracks this cohort's offending rates through to their last entry in the table at age 40 to 44 years in the period 2010 to 2014 with a rate of 20.61.

Note that our birth cohorts span a century: consider that a person who was aged 50 when arrested in 1960 was born in the year 1910. However, note also that this means the data set has a reduced number of data points for cohorts at either end (as described by O’Brien 2019a). This is because, and to continue the example, while we have arrest data for the older age groups of the 1910 birth cohort, we do not have it for the younger age groups. Similar truncation of the data takes place for recent years: arrest information on the 1990 birth cohort, for instance, was only available up to age 24 by the year 2014.

### The APC analysis

As suggested earlier, there is no consensus over which approach to APC analysis is best. This reflects the thorniness of the problem, which is that age, period, and cohort are influenced by each other, which makes disentangling them difficult. This is known as the identification problem (Fu 2018, Bell 2020). The three APC factors are linearly dependent, meaning that if two of them are known then the third can be calculated. For example, for a person who was aged 30 in 1990, it can be deduced that they are part of the 1960 birth cohort. However, the result of linear dependency is that for any given period (here gauged in years), the individual effects of age, period and cohort on the arrest rates cannot be distinguished.

Methods that partially overcome the identification problem have been proposed by O’Brien (2014, 2015, 2019a,b). These restrict the possible solutions to a smaller set, by mathematically introducing ‘known’ theory. Specifically, there has been quite a lot of previous research on age and crime (Farrington 1986, Farrington, Loeber and Jolliffe 2008), and studies showing age-related changes in offending during recent decades of declining crime also tend to show that the adolescent age-peak has remained (Matthews 2014, Farrell, Laycock and Tilley 2015, Matthews and Minton 2017, Payne, Brown and Broadhurst 2018). O’Brien (2019a) reviews the age-related criminal career literature issue at length and concludes that it is reasonable to make assumptions about the expected values of the Age effect, and we follow this practice of ‘fixing’ the Age parameters in our models. By restricting the values of age using this ‘known’ aspect, it becomes easier to disentangle the period and cohort effects. The peak age of motor vehicle theft offending is in the 15–19 year old age group, which in the present context is one age group younger than that for homicide as used by O’Brien (2019a).

The analysis was undertaken in R utilising Fu’s APC package (Fu 2018) and the constrained method and the S-constraint method (O’Brien 2014, O’Brien 2019b). The ‘constrained method’ places a constraint by fixing an exact relationship between two of the parameter values. This forces us to select a single model. An example of a constraint is if two period effects, say, 1980–1985 and 1985–1990, are assumed to be the same. This constraint means that a single model (i.e. set of APC estimates) can be determined from what is otherwise a mathematically infinite number of models. Often, however, the relationship cannot be described so precisely. For example, we may know that there is an age-related peak in offending, but the gradients on either side cannot be specified. To overcome this, the ‘S constraint method’ adds flexibility. A parameter, S, is used to adjust the model selected, so that the boundaries of the theory can be found. In our hypothetical example, the two period effects, 1980–1985 and 1985–1990, might reasonably be assumed to be within 5 percent of each other. The result of our enhanced S-constraint method is a set of possible solutions rather than a single answer. However, while the answer from the S-constraint method is less *precise* (it is no longer a point estimate), it is more *accurate* (it is more likely to contain the true value).

Here, and following O’Brien (2019b), the basis of the analysis is the Age-Period-Cohort Multiple Classification (APCMC) model of equation

1 $$y_{ij} = \mu + \alpha_{i} + \pi_{j} + \chi_{I - i + j} + \in_{ij} .$$

Where $y_{ij}$ is the theft rate for the ith age group in the jth period (where i and j are 1,2,3…). $\alpha_{i}$ is the coefficient for the ith age group. $\pi_{j}$ is the coefficient for the jth period. $\chi_{I - i + j}$ is the coefficient for the corresponding cohort and $\in_{ij}$ is the error term.

This is the APCMC model that, as noted above, would potentially identify an infinite number of possible solutions that is, combinations of age, period, and cohort effects, without inducing any known theory. The basis of our analysis was to use this APCMC model to explore relationships in the data by introducing the following constraints/theory; (1) Specifying a late-teen peaked age curve (2) Inducing a period effect to represent effectiveness of Steering locks, (3) Inducing a period effect to represent effectiveness of immobilisers, and (4) Inducing a trend which shows that cohort of birth was not significant (a cohort-zero-linear trend).

Hence our main analysis followed the S-constraint approach pioneered by O’Brien (2019a,b). However, we introduced two modifications. The first was to amalgamate multiple constraints into a single multifaceted constraint. O’Brien’s analysis made two key assumptions relating to the shape of the age-crime curve. The first was that the age coefficients were the same for the two age groups either side of the peak age. The second was that the age coefficient declined monotonically after the peak age group. O’Brien conducted a separate analysis of each whereas we combined both constraints into a single analysis to specify S, as described in the Appendix.

The second modification to the S-constraint approach relates to the trade-offs between precision and accuracy that we described above. O’Brien selected single values of S which necessarily produced single-value estimates (O’Brien 2019b). Single estimates are precise, and could be interpreted as implying that values in-between are also plausible. We produced a range of values of S, which therefore produced a range of values of estimates. The advantage of this, we suggest, is that the results are more accurate (the true value is more likely to be within the set) even though a range is less precise. We include further details in the appendix.

We also explored the possibility of making evidence-based assumptions about the cohort effect, as outlined by O’Brien (2019a). Our study differs in the way this aspect of the approach is adopted, which reflects the fact that we are studying motor vehicle theft rather than homicide. We explored the possibility of identifying the number of vehicles without electronic immobilizers over time as a measure of target availability. However, we were only able to identify this data for some years and not others. This meant that, while we were unable to undertake such an analysis in full, the partial dataset was explored to assist in the explanation of the cohort effects, and this is described in the findings, below.

## Findings

**Fig. 2:**
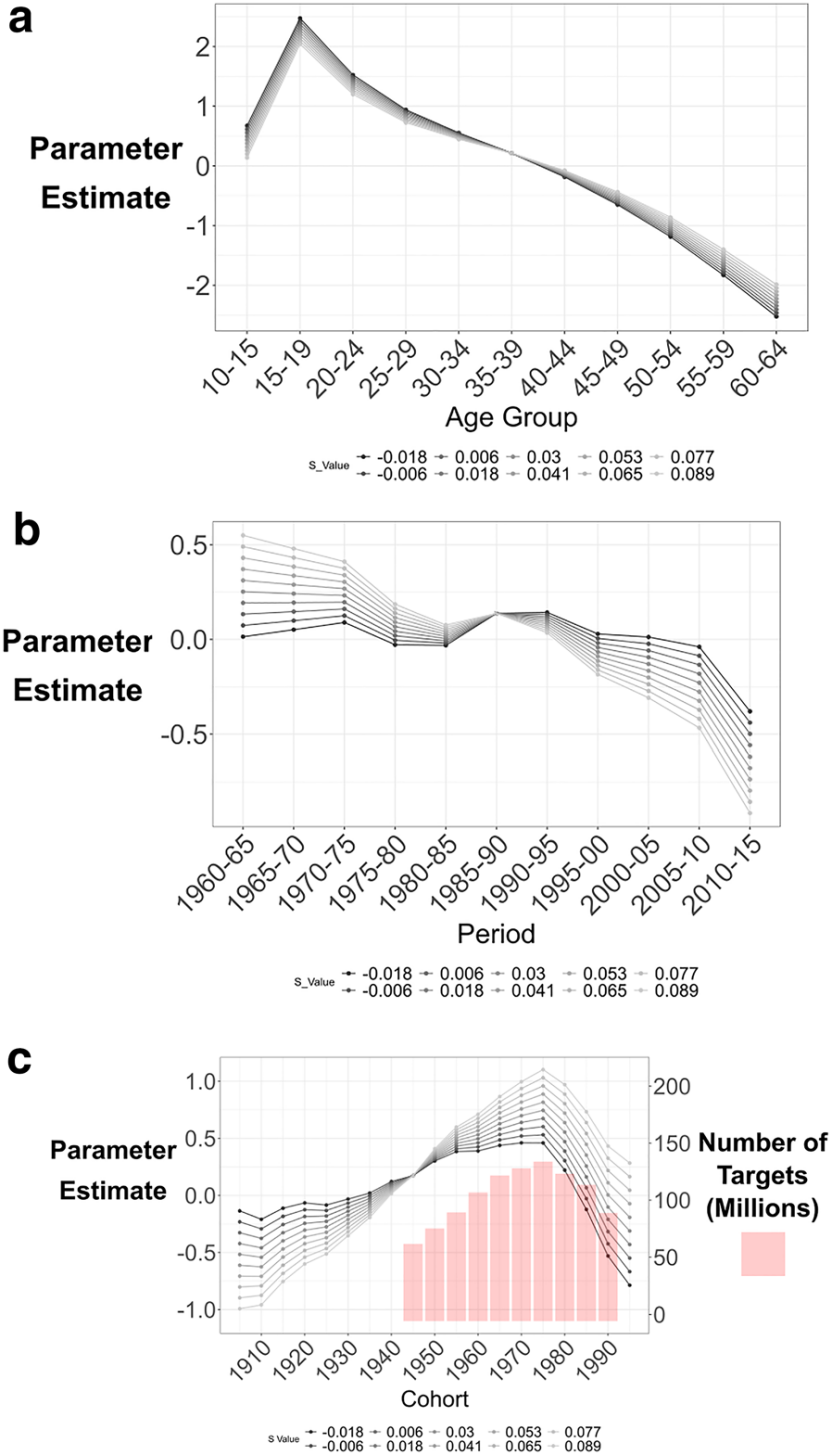
**a** Age parameters from the S-constraint model. **b** Period parameters from the S-constraint model. **c** Cohort parameters from the S-constraint model, Red bars indicate the number of potential targets to which cohorts had access in their peak offending years

The various elements of the analysis yielded largely similar results. Consequently, for brevity, here we discuss the findings for the Age S-constraint method, with those for the constrained methods and the cohort zero-linear-trend included in the Appendix. The sub-set of possible solutions for each parameter that was identified by the Age S-constraint method is represented in Fig. 2a–c (and the figures in the appendix show equivalent graphs for the additional analyses). Each of Fig. 2a-c shows a set of lines representing an even spread through the values of S that fell within the constraint. A close arrangement of lines, with similar patterns, indicates strong evidence that a true relationship has been specified between that parameter and the arrest rates. Findings for each parameter will be presented in turn.

### Age

To re-state—because it is central to understanding the findings—the Age parameter was given some ‘known’ characteristics based on previous research. This is reflected in the findings. Vehicle theft offending was taken to peak at the 15 to 19-years old category, and to have gradients either side of the peak that were within the range of the original arrest rate data gradients. It was further constrained by the specification of a monotonic decline from the peak. Although utilising the original age distribution of arrests as a basis for the constraint could introduce cohort effects, we would expect them to be minimal because we used the age data from all of the years in the study.

Figure 2a show the Age peak and a steady monotonic decline as age increases. The range of values for the age parameters is narrow and all of the values of S result in a very similar pattern.

### Period

Findings for the period parameter, in Fig. 2b, demonstrate a reasonably narrow range of parameter estimates (that is, the lines are clustered rather than spread out). It also exhibits similar patterns across all values of S.

Among the range of parameter estimates, the general trend is for a decreasing period effect, with three notable patterns. First, there was a steep decline after the early 1970s. Second, there was an incline to the early 1980s. Third, there was a decline from the early 1990s onwards.

### Cohort

The close clustering of the lines on the cohort parameter graph at Fig. 2c indicates a relatively consistent picture across all values of S. Cohort effects appear to put upward pressure on motor vehicle theft until the 1975 cohort, and to decline thereafter. There is some variation between models in the gradient to the peak and in whether it is monotonic. All these models suggest that cohorts prior to around 1975 were increasingly likely to commit motor vehicle theft as time went on. The 1975 and subsequent cohorts were less likely to commit car crime, and the drop was steeper than the gradual climb to 1975.

The vertical bars shown in Fig. 2c represent the estimated number of available target vehicles (those without an electronic immobilizer) encountered by a cohort in their peak offending years. The estimate was derived as the number of passenger vehicles on the road (Bureau of Transportation Statistics 2017) less those with an immobilizer (from Morgan et al. 2016, as described in the Appendix). The bars show the estimate of non-immobilized cars in each cohort’s peak offending years. This suggests that the cohort parameters track the level of targets available in the peak offending years of cohorts.

## Discussion

Figure 2a-c show that the age effect had a greater influence on the arrest rate than the cohort effect, which in turn is greater than the period effect, across all values of S for the age constraint. This means that someone’s age has more effect on their vehicle theft rate than the period in which they were born, which in turn has a stronger effect than the period of time they currently find themselves in.

There is reason to be confident that the period and cohort effects identified here accurately represent these aspects of change relating to motor vehicle theft. The reason for that confidence is that the well-known age-crime curve was reproduced, through inducement, in the findings for the age effect. As all variables are fixed in relation to one another, replicating a single variable in a way consistent with established evidence strongly indicates that the patterns for the other two variables are also consistent with that evidence. In short if the spread of the age-crime curves contains the correct representation, then the resulting spread of patterns from the period and cohort effects will also contain the correct respective patterns.

The findings for the period parameter—downward pressure on theft in the 1970s, upward in the 1980s, and increasing downward pressure from the early 1990s onwards—correspond with the theft rate trends overall (shown in Fig. 1). That is, the characteristics of the period parameters correspond with potential effects of the major vehicle security initiatives introduced during the early 1970s and the 1990s that were discussed earlier. However, it is through the cohort effects, discussed next, that the key long-term effects of the security improvements appear to operate.

The cohort parameter findings suggest decreasing downward pressure from cohort effects for most of the first half of the twentieth century, upwards pressure in the post-war period until the mid-1970s. This was followed by a reduction in the strength of that upward pressure which becomes downward pressure from around the late 1980s to the 1990s. The manner in which the cohort effect tracked target availability in a cohorts peak offending years, shown in Fig. 2c, suggests that the introduction of immobilizers, that is, the constriction of targets, affects the rate of offending for a cohort rather than just at the period they were introduced. That is, the evidence suggests that the period effect, which operates differentially by age, may produce a cohort effect across the life-course of these offenders. A potential steering lock effect upon cohorts was less pronounced, with some difference in the rate of change between the parameter and the target estimates for the 1945 and 1950 cohort, but it is hard to discern and so should not be over-interpreted.

In their work on homicide, O’Brien and colleagues observed that “Determining that cohorts vary is different than explaining why these effects occur. One way of considering this question is to view the historical periods in which cohorts are born as opportunity structures, settings that provide both opportunities and constraints for members of a birth cohort … These opportunities and constraints may result from historical events…” (O’Brien, Stockard and Isaacson 1999; 1063). This fit wells with the interpretation of cohort effects as significantly influenced by changes to the crime opportunity structure in relation to motor vehicle theft trends. The present findings are consistent with opportunity-reducing crime prevention measures reduced the rate of offending over the life-course for cohorts spanning many years.

There is scope to unpack the mechanism by which the cohort effect occurred, which we suggest was as follows. From the early 1990s onwards, it gradually became less easy for adolescents to begin offending as an increasing proportion of vehicles became secure (as secure new vehicles were purchased, and insecure old ones scrapped). Potential young offenders did not have the skill or experience to overcome the new vehicle security technology, particularly electronic immobilizers. The result was a reduced rate of offending, not just as a short-term period effect when they were adolescents, but among cohorts which experienced lower rates of offending as they aged through the life-course. The rate of offending among subsequent cohorts decreased further over time as improved vehicle security became more prevalent and continued to improve, fewer adolescents experiencing criminal career onset and continuance.

We have given the nature of the age-effect relatively little attention. An interpretation consistent with crime opportunity theories is that certain age groups are more likely to be in situations, due to physical ability, lifestyle and environmental factors, which allow or encourage them to avail themselves of a greater level of prevailing crime opportunities. Although not the present focus, this could explain why motor vehicle theft is historically associated with lower income areas: lower income areas are populated more by vehicles with lower-level, more familiar security, an interpretation consistent with changes in patterns of vehicle theft as it declined elsewhere in north America (Hodgkinson et al. 2016).

Can the APC effects for motor vehicle theft be reconciled with those for homicide as identified in O’Brien (2019a)? In terms of APC effects, the findings appear quite similar. If over-time variation in the two crime types is connected, there is a need for further research here. It is possible that homicide, which is statistically relatively rare, varied at least partly because of variation in the vehicle theft rate. This could occur if offenders who successfully completed the easier early-career crimes, such as vehicle theft, progressed shortly afterwards to more serious crime including gun carrying (White, Loeber and Farrington. 2008). A multiplier effect could have occured when vehicle theft preceded homicide caused by dangerous driving, or when a stolen vehicle was used in the commission of further crimes including homicide. These possibilities have been posited as the debut crime and keystone crime hypothesis respectively (Farrell et al. 2011). Additional APC analysis for other crime types, countries or areas may shed further light on such issues.

### Study limitations

This APC analysis uses constraints, and as such can only be as good as the evidence that is used to identify those constraints (O’Brien 2019a, Bell 2020). However, the key assumption underlying the constraint was the shape of the age-crime curve which, as discussed earlier, is considered well established.

A potential limitation of the present analysis was the grouping of age and period data. This could make some features in the period variable curves less pronounced. The estimation for the targets in the cohort discussion is limited by the use of grouped data, the absence of information on vehicle models and the non-random introduction of immobilizers. In addition, the reduced number of data points at either end of the cohort range, as discussed in relation to the arrest data, could affect the findings if the missing data points were systematically different to those included, though there is no reason to expect this occurs here.

We have proposed that a strength of the present study is the novel adaptation of O’Brien’s S-constraint approach. This offers, we think, an opportunity for further research to gauge the precise nature and extent of any resulting difference. However, we recognise that the absence of such a metric here could be considered a limitation.

This study sought to provide transparency with respect to method, in order that readers who wish to explore other possible interpretations of events and hypotheses, can do so. However, some readers will likely suggest the study should have considered alternate potential explanations for APC effects identified here. Our response is that other crime drop hypotheses were not considered because they have been largely falsified by other means in previous research (Farrell 2013, Farrell et al. 2014, Tilley et al. 2018), summarised as.

“Of course, it is still possible that other factors also played a role in the crime drop, but this seems increasingly unlikely in the face of evidence supporting the security hypothesis, and largely refuting other hypotheses, that has emerged in recent years.” (Farrell and Birks 2020; 3).

## Conclusions

This study adopted O’Brien’s S-constraint approach as state-of-the-art in APC analysis. We offered two methodological refinements to the approach. The first allowed us to put more constraints into a single analysis, producing, we suggest, more accurate findings. The second returned a plausible range of models rather than a single estimate, which is more likely to contain the real values than a point estimate. The overall effect was to hone the possible set of solutions.

In orientation, this study has sought to heed Bell’s (2020) clarion call for conceptual clarity in the application of APC. Here, and following O’Brien (2019a), our APC analysis was facilitated by the assumption of a known relationship between age and vehicle theft offending, which previous research indicates is a very reasonable assumption. The reproduction of the known age-crime curve in the age-parameter findings gives confidence in the accuracy of the findings relating to period and cohort effects. That is, overall, there is sound methodological reason to be confident in the findings. From this platform, we offer substantive conclusions in two main areas. The first relates to the international crime drop. The second relates to the nature of crime and offending more generally.

With respect to the international crime drop, the study provides new and potentially important information about the nature and causes of the trends in motor vehicle theft over the last half century. The findings lend weight to the security hypothesis, that is, to the proposition that vehicle security improvements were primarily responsible for the long-term decline in vehicle theft in the United States. Within the canon of crime drop research, this finding is a notable addition to the growing evidence that the major long-term reductions in crimes across many countries were largely due to security improvements.

We also conclude that the study facilitates new insight into adolescent and life-course offending. Not only was the rate of adolescent offending reduced by vehicle security improvements, but so too was the subsequent rate of offending among those cohorts affected. This leads us to conclude that the prevailing level of crime opportunities during adolescence is a central determinant of the level of offending in a birth cohort as it ages. It may suggest the need for a form of developmental crime science, which would be a radically different perspective to developmental criminology’s view of offending as determined largely by earlier childhood experiences.

## Figures and Tables

**Fig. 3:**
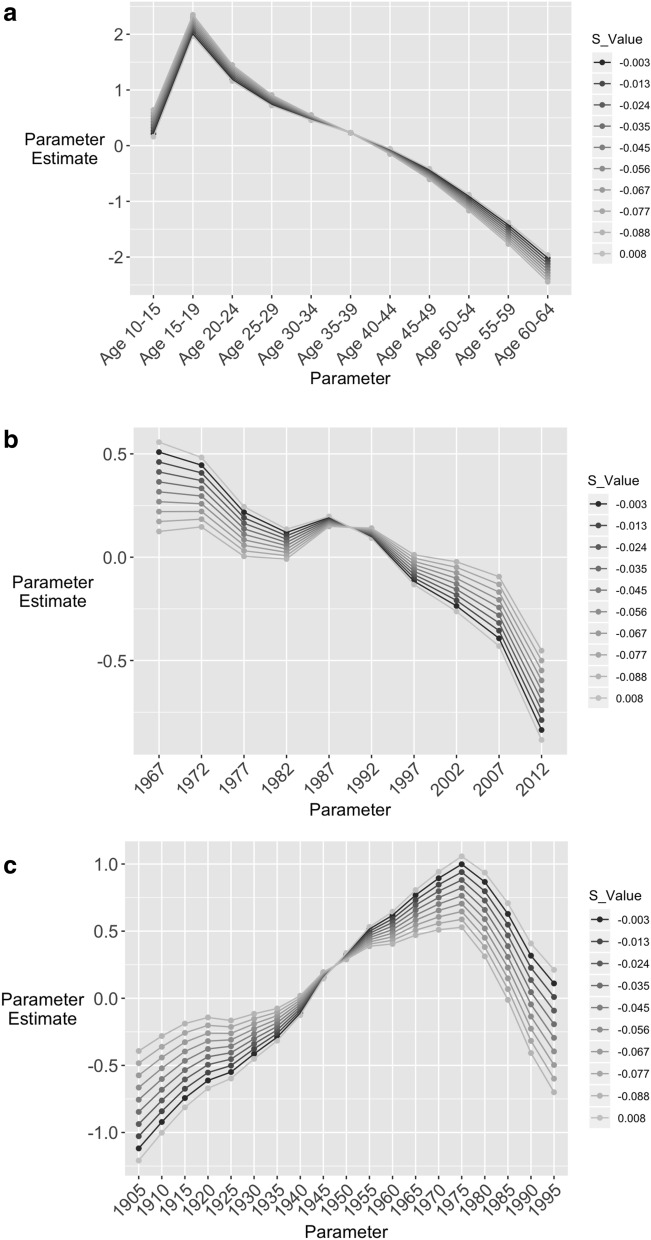
**a** Age parameters from the S-constraint model using data from 1965 to 2010 only (S-constraint from the peak age constraint APC model of motor vehicle arrests). **b** Period parameters from the S-constraint model using data from 1965 to 2010 only y (S-constraint from the peak age constraint APC model of motor vehicle arrests). **c** Cohort parameters from the S-constraint model using data from 1965 to 2010 only (S-constraint from the peak age constraint APC model of motor vehicle theft arrests)

**Fig. 4:**
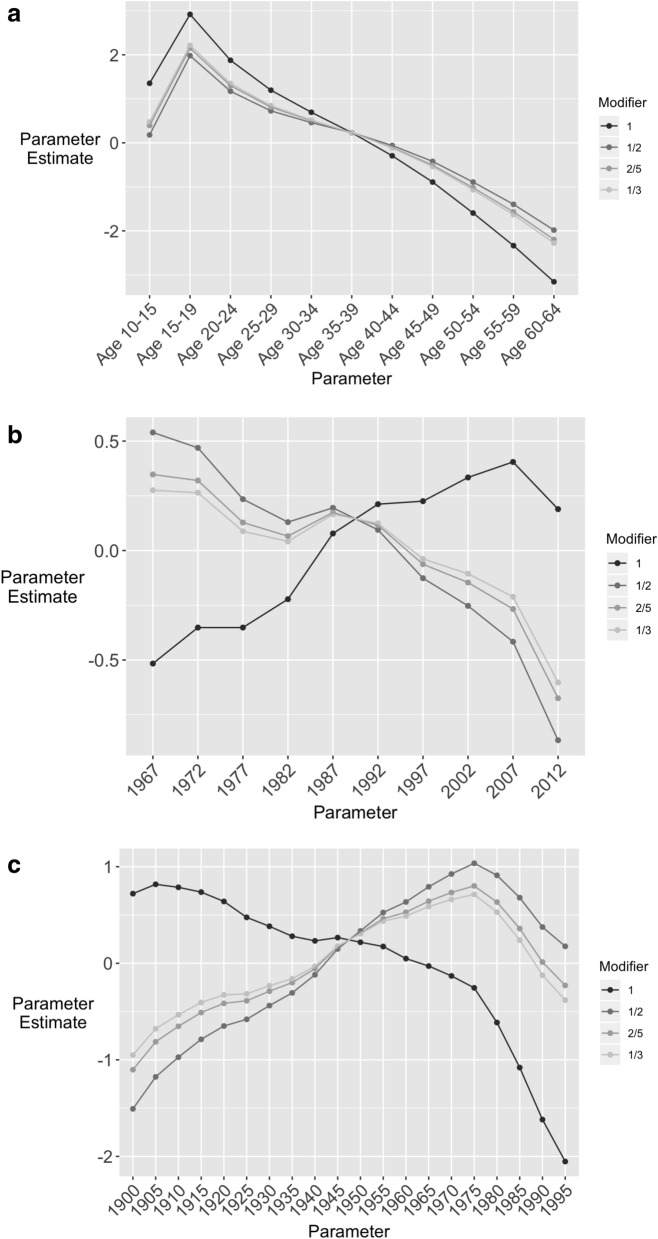
**a** Age parameters from the constrained model using data from 1965 to 2010 only (Period constraint from early to mid-1970’s). **b** Period parameters from the constrained model using data from 1965 to 2010 only (Period constraint from early to mid-1970’s). **c** Cohort parameters from the constrained model using data from 1965 to 2010 only (Period constraint from early to mid-1970’s)

**Fig. 5:**
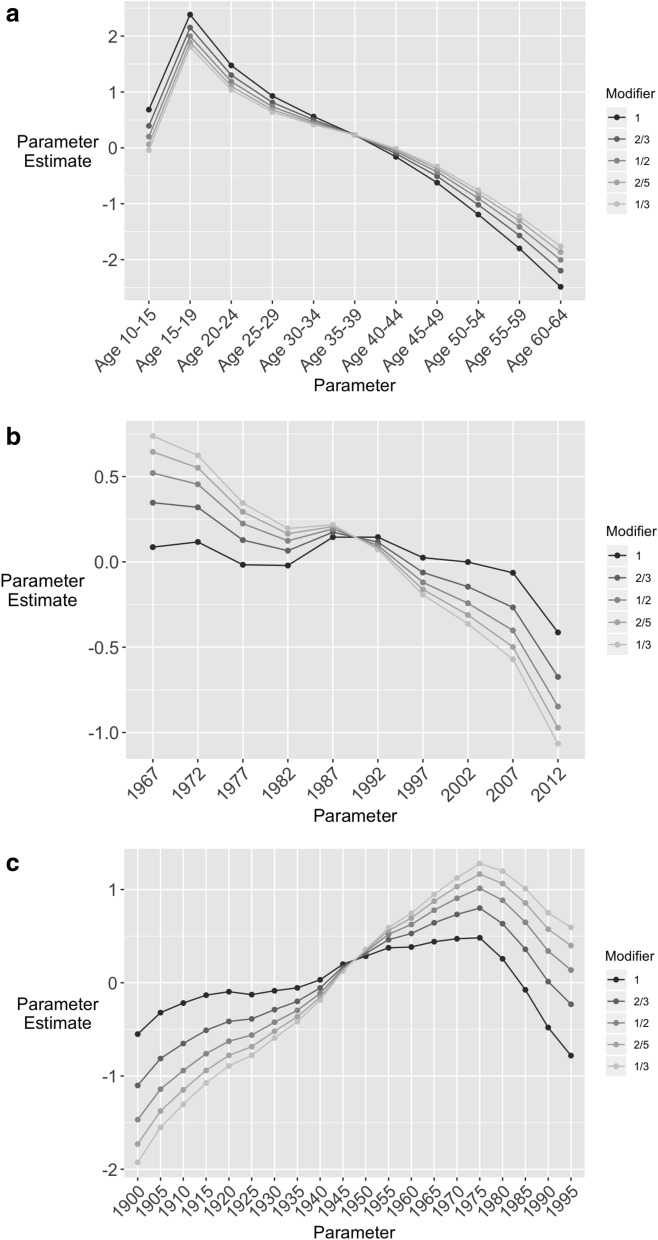
**a** Age parameters from the constrained model using data from 1965 to 2010 only (Period constraint from late 1980’s to early 1990s). **b** Period parameters from the constrained model using data from 1965 to 2010 only (Period constraint from late 1980’s to early 1990s). **c** Cohort parameters from the constrained model using data from 1965 to 2010 only (Period constraint from late 1980’s to early 1990s)

**Fig. 6:**
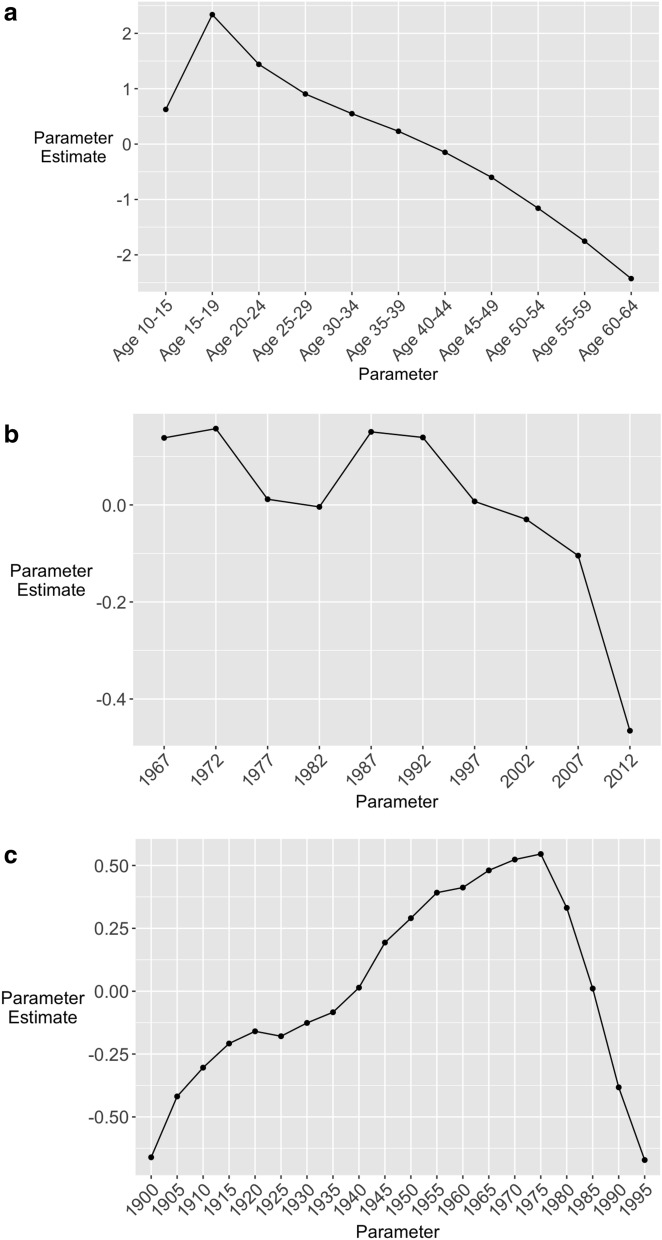
**a** Age parameters from the S-constraint model using data from 1965 to 2010 only (S-constraint from the Minimum cohort range constraint APC model of motor vehicle arrests). **b** Period parameters from the S-constraint model using data from 1965 to 2010 only (S-constraint from the Minimum cohort range APC model of motor vehicle arrests). **c** Cohort parameters from the S-constraint model using data from 1965 to 2010 only (S-constraint from the Minimum cohort range APC model of motor vehicle theft arrests)

**Fig. 7:**
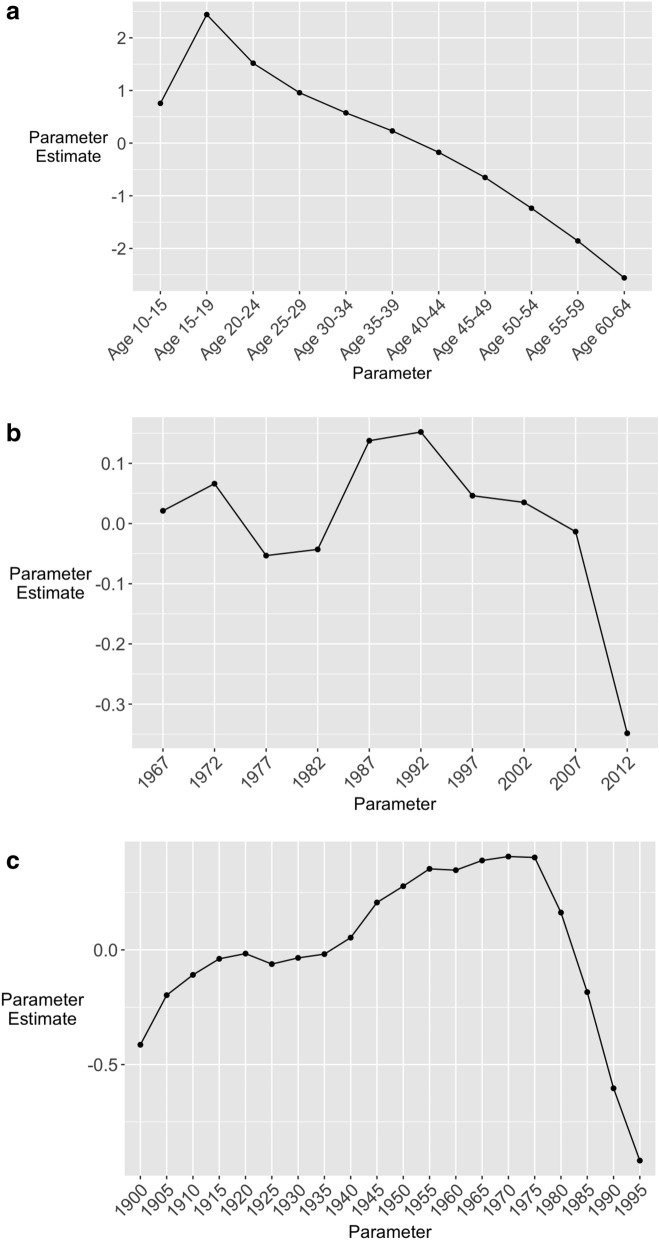
**a** Age parameters from the S-constraint model using data from 1965 to 2010 only (S-constraint from the Minimum cohort effect constraint APC model of motor vehicle arrests). **b** Period parameters from the S-constraint model using data from 1965 to 2010 only (S-constraint from the Minimum cohort effect APC model of motor vehicle arrests). **c** Cohort parameters from the S-constraint model using data from 1965 to 2010 only (S-constraint from the Minimum cohort effect APC model of motor vehicle theft arrests)

## Data Availability

The data are publicly available.

## Appendix

This appendix describes further aspects of the main analysis plus supporting analyses.

### Further details of main analysis

As noted, the main analysis largely followed O’Brien (2019a). We extended it by using a search algorithm to identify a selection of solutions rather than one solution to fit the theoretical constraints. The steps in the analysis were:

1. We used the Fu R Function ‘apclinfit’ to fit APC models to the data (see Fu 2018; p37). Separate models were fitted to explore theoretical constraints of (a) known age curve and fixing period effects, (b) 80’s, and (c) 70’s.
  - apclinfit(r = data, apcmodel = ”APC”, header = T, transform = ”log”, apc = 2, lindex = c(5,6), lvalue = c(−1,2))).
  - apclinfit(r = data, apcmodel = ”APC”, header = T, transform = ”log”, apc = 2, lindex = c(5,6), lvalue = c(−1,2))).
  - apclinfit(r = data, apcmodel = ”APC”, header = T, transform = ”log”, apc = 2, lindex = c(2,3), lvalue = c(−1,2)).

With the resulting solutions to the Age-Period-Cohort multiple classification (APCMC) model, the null vector was constructed, which allowed us to use the S-constraint method.

2. The Fu Function apcmat was used to create an effect coded matrix.
3. Following the equations in O’Brien’s work (2014, Appendix 2.2), the null vector was created. The null vector was validated by matrix multiplication with the effect matrix.
4. An extended null vector (EnV) was created following O’Brien (2014; p31). The EnV was used to ensure the reference category was included in the vector.
5. The EnV was used to modify one solution to find another solution using a variable S using the following equation (O’Brien 2014; 216):
  $$b_{c}^{0} = b_{c1}^{0} + s\nu ,\,s\text{ } \in {\mathbb{Z}}$$
  where $b_{c1}^{0}$ is an already discovered solution to the APCMC equation, $b_{c}^{0}$ is a new solution to the APCMC equation, $\nu$ is the extended null vector and s is a scalar.
6. A search (brute force) approach was used to compute $b_{c}^{0}$ over a given range of S. This was searched to isolate values of s with a corresponding $b_{c}^{0}$ fitting a given theoretical pattern. $b_{c}^{0}$ vectors were checked with simple inequalities (e.g. age2 > age3 > age4 > age5) to ensure they conformed to the theoretical based pattern. Results were filtered to leave only those values of S that conformed to the theory. This allowed the model to return a relatively narrow range of possible solutions, rather than a single point estimate. The benefit being that the real solution would more likely be in that narrow range than equal to the single point estimate. The constraints used to specify the age curve were:
  - That the peak offending rate was at age 2, so 15 to 19 years of age.
  - The relative difference in rates either side of the peak, with respect to the peak, were constrained so that they existed in the range already present in the data.
  - There was a monotonic decline from the peak age onwards.

### Issue of partial missing data 1960–1963

We replicated the S-constraint analysis described in the main text, but excluded the 1960 to 1963 arrest data. This was to check that the findings were not skewed by our replacement of missing values for the pre-teen and post-55 age groups, which were absent from the FBI arrest data for those years. The findings of the replication, shown as Fig. 3a–c, are almost identical to those in the main text. Hence we concluded that no significant error was introduced.

### Constrained method for period effects

Constraints were placed on the period parameters to induce the effects expected by the introduction of security measures. This was completed using the Fu R packages and with an array of strength associations. The graphs at Fig. 4a-c show a period effect for the seventies, and Fig. 5a-c show a period effect into the early nineties. A modifier of 1 for the 1970s constraint (setting the period effects as identical) produced a plausible age curve but cohort and period effects that differed from the main findings. Given the stark contrast of cohort parameters with all of the other models we conclude that this is a solitary point of weak evidence against a decline in period effects across the seventies. Both of these constraints produce age curves which are consistent with accepted theory. Additionally they both produce each other’s decline across an array of strength associations. We conclude from this that there is good evidence for period effects consistent with the major vehicle security interventions from this analysis (Figs. 6a-c and 7a-c).

### Cohort zero-linear-trend

To test the robustness of the cohort effects described in the main text, we used the S-constraint process to overtly minimise the cohort effects. This was achieved by optimising the value of S for two equations. The first was finding the minimum range of values of the cohort parameters, which would mean that the cohorts had similar effects over time. The second optimisation was to find the set of cohort parameters that were closest to zero (analogous to the root mean square error to the line y = 0). Optimisation was achieved through a brute force search of a range of values of S. The values of S which optimised these two equations were different but relatively close (0.215(3dp) and −0.275(3dp)). These values of S were then used to generate the APC parameter graphs shown here as Figs. 6a-c and 7a-c. What is striking about these graphs is that the cohort patterns still contain a similar pattern and that the cohort parameters, despite being minimised, are still stronger than the period parameters.

### Target estimation

The estimate of the number of targets—vehicles without immobilizers—used in the main text was derived as the multiple of the number of passenger vehicles in the US (Bureau of Transportation Statistics 2017) and an estimate of the percentage of cars with an immobilizer (from Morgan et al. 2016). Morgan et al. 2016 estimated immobilizer penetration using information from the National Institute for Highway Safety data and vehicle fleet age, and concluded that it was conservative, that is, likely an underestimate. We could not find the calculations in their text but the estimates were presented in a chart (Morgan et al. 2016, Fig. 23) and available in an accompanying spread sheet.
